# Downregulation of Six MicroRNAs Is Associated with Advanced Stage, Lymph Node Metastasis and Poor Prognosis in Small Cell Carcinoma of the Cervix

**DOI:** 10.1371/journal.pone.0033762

**Published:** 2012-03-16

**Authors:** Long Huang, Jia-Xin Lin, Yan-Hong Yu, Mei-Yin Zhang, Hui-Yun Wang, Min Zheng

**Affiliations:** 1 State Key Laboratory of Oncology in Southern China, Cancer Center, Sun Yat-sen University, Guangzhou, People's Republic of China; 2 Department of Gynecology, Cancer Center, Sun Yat-sen University, Guangzhou, People's Republic of China; 3 Department of Obstetrics and Gynecology, Nanfang Hospital, Southern Medical University, Guangzhou, People's Republic of China; Kyushu Institute of Technology, Japan

## Abstract

**Background:**

Small cell carcinoma of the cervix (SCCC) is very rare, and due to the long time period required to recruit sufficient numbers of patients, there is a paucity of information regarding the prognostic factors associated with survival. MicroRNAs (miRNAs) have been used as cancer-related biomarkers in a variety of tumor types, and the objective of this study was to determine whether microRNA expression profiles can predict clinical outcome in SCCC.

**Methodology/Principal Findings:**

Forty-four patients with SCCC who underwent radical hysterectomy between January 2000 and October 2009 were enrolled. Using the GeneCopoeia All-in-One™ Customized Human qPCR Primer Array, the expression profiles of 30 miRNAs associated with tumor metastasis was obtained from the formalin-fixed paraffin embedded samples of all 44 patients. Seven miRNAs, has-let-7c, has-miR-10b, has-miR-100, has-miR-125b, has-miR-143, has-miR-145 and has-miR-199a-5p were significantly down-regulated in advanced stage SCCCpatients (FIGO IB2-IV) compared to early stage SCCC patients (FIGOIB1). Among, downregulation of six miRNAs, has-let-7c, has-miR-100, has-miR-125b, has-miR-143, has-miR-145 and has-miR-199a-5p were significantly associated with lymph node metastasis and reduced survival in SCCC. Kaplan–Meier survival analyses revealed that SCCC patients with low expression of has-miR-100 (*P* = 0.019) and has-miR-125b (*P* = 0.020) projected a significant tendency towards poorer prognosis.

**Conclusions/Significance:**

This study demonstrates that downregulation of 7 miRNA associated with advanced stage, 6 miRNAs with metastasis and 2 with poor prognosis in SCCC. Functional analysis of these miRNAs may enhance our understanding of SCCC, as altered expression of specific miRNAs may regulate the metastatic pathway and provide novel targets for therapy.

## Introduction

Neuroendocrine small cell cervical carcinoma (SCCC) is an aggressive, rare form of cervical cancer, accounting for less than 3% of all cervical cancers [1]–[3]. SCCC is characterized by a high incidence of early nodal and distant metastases, resulting in a poorer prognosis than other subtypes of cervical cancer [4]–[6]. Previous studies have reported that 60–82% of SCCC patients have lymph-vascular space infiltration or pelvic lymph node metastasis at diagnosis [7]–[9]. Additionally, SCCC exhibits a propensity for rapid distant metastasis via the bloodstream to various sites including the liver, lung, brain, bone, pancreas and lymph nodes, which results in treatment failure in most cases [8]–[11]. Recurrences usually occur within 2 years, and most patients die as a result of early metastasis. It is important to identify the factors responsible for the survival of metastases in order to improve treatment strategies for SCCC. However, due the rarity and the long time period required to enroll a sufficient number of patients, most studies on SCCC are comprised of small series or case reports, making it difficult to determine the optimal therapy.

MicroRNAs (miRNAs) are noncoding RNAs 18 to 25 nucleotides in length [12]. The effects of miRNAs are mediated by binding to target mRNAs, to either suppress mRNA translation or degrade miRNA-bound mRNA [13]. Currently, more than several hundred unique mature human miRNAs are known, and many are involved in tumorigenesis, acting either as oncogenes [14] or tumor suppressors [15], [16]. Aberrant expression of miRNAs has also been linked to cancer [17], [18], suggesting that miRNAs potentially represent prognostic markers, and leading to the use of miRNA profiling for the diagnosis and prognosis of specific cancers. It has been shown that miRNAs are involved in every type of cancer examined to date; however, the expression of miRNAs in SCCC has not been investigated.

In this study, miRNA qPCR arrays were performed on 44 SCCC samples, as we hypothesized that investigation of miRNA profiles would provide more information on SCCC, an inadequately understood disease with a poor prognosis.

## Materials and Methods

### Ethics Statement

All patients agreed to participate in the study and gave written informed consent. This study was approved by the medical ethics committee of Cancer Center of Sun Yat-Sen University and complied with the Declaration of Helsinki.

### Samples and Cases

Formalin-fixed paraffin-embedded tissues (FFPETs) from 44 SCCC patients undergoing radical hysterectomy between January 2000 and October 2009 were obtained from the Cancer Center, Sun Yat-SenUniversity, Guangzhou, P.R.China. The patients had not received any preoperative radiotherapy and/or chemotherapy, and the SCCC diagnosis was confirmed by pathology. All patients were staged according to the International Federation of Gynecology and Obstetrics (FIGO) staging system for cervical cancer.

### MicroRNA extraction and reverse transcription

The Leica DMLA system (CTRMIC from Leica, Germany) was used to microdissect the tumor cells and the stromal cells from the FFPET sections. Ten sections were used to obtain sufficient RNA for reverse transcription polymerase chain reaction (RT-PCR), Processing of the total RNA began immediately following microdissect. Total RNA was isolated using the miRNeasy FFPE Kit (Qiagen, Hilden, Germany) according to the manufacturer's instructions, and the quality and concentration of RNA was determined using a Nanodrop 1000 (Thermo, Wilmington, DE, USA). MicroRNAs were isolated from total RNA using the All-in-One™ miRNA qRT-PCR Detection Kit (GeneCopoeia, Rockville, Md, USA), according to the manufacturer's instructions, by reverse-transcription of mature miRNAs using Poly-A polymerase with an oligo-dT adaptor.

### MicroRNA qPCR Array

The All-in-One™ Customized Human qPCR Primer Array (HAQPA-1103-01-24D; GeneCopoeia Inc, Rockville, Md, USA) 96-well-qPCR plate was used for this study. Each array is a panel of validated, optimized qPCR primers for 30 miRNAs closely associated with tumor metastasis, and the housekeeping genes *RNU6B* and *SNORD44*, which are used as reference factors to normalize the expression results. Each well contains a forward primer for the mature miRNA sequence and a universal adaptor reverse primer cross-linked to the 96-well plate. The qPCR Primer Array was performed in 20 µl reaction volumes containing 1 µl reverse transcription product, according to the manufacturer's instructions using SYBR Green detection on the ABI 7900HT instrument (Applied Biosystems, Carlsbad, CA, USA).

### Confirmed all PCR products by sequencing

TA clone was performed according to the protocols with minor modifications of TOPO TA Cloneing Kit for sequencing (Invitrogen). Briefly, purify the PCR products with PCR products purification kit (QIAGEN) first. Then pool 1 µl TA vector (TOPO-TA cloning kit, Invitrogen), 1 µl 10×_ ligation buffer, 5 µl PCR products, 1 µl Ligase into one tube and add water to make the final volume 10 µl, Ligate at 14_ overnight. The next day, Combine one tube of One-Shot TOP10 competent cells (Invitrogen) with 2 µl of the previous ligation products and use X-gal/IPTG to screen white colonies as colonies with insert. Final, pick up 10 white colonies to make plasmid DNA and sent to Invitrogen (Guangzhou, China) for DNA sequencing.

### Follow-Up

The patients were followed every 3 months for the first year and then every 6 months for the next 2 years and finally annually, thereafter. The total follow-up period was defined as the time from diagnosis to the date of death or the last date censused if patients were still alive. Censoring refers to the patients who did not diagnosis in our hospital and can not provide actual diagnose time. There are all 42 patients that is included in survival data analysis. The last follow-up was carried out in December 2010, with a mean observation period of 23.6 months (range: 2–70 months) and 13 cancer-related deaths.

### Statistical Analysis

The miRNA expression levels were analyzed using the GeneCopoeia online Data Analysis System (www.genecopoeia.com.cn/product/qpcr/analyse/). Using the relative quantification (RQ) equation, where ΔΔCT = (CT_miRNA_−CT_RNU6B+SNORD44/2_) Mean _metastasis_−(CT_miRNA_−CT_RNU6B+SNORD44/2_) Mean _no-metastasis_). Cluster analysis was performed using the MultiExperiment Viewer 4.2 software (http://mev.tm4.org). Statistical analysis was carried out using SPSS Version 13.0 (SPSS Inc., Chicago, IL, USA). Survival curves were estimated by the Kaplan–Meier method, and compared using the log-rank test. Parameters that were significantly related to survival in univariate analysis were entered into the multivariate analysis. With a Cox proportional hazard model, multivariate analysis was done to identify the independent prognostic factors. Receiver operating characteristic (ROC) curve analysis was employed to determine whether the miRNAs found are really good candidates to discriminate early from advanced tumour stages and presence or absence of lymph node metastasis. Two-sided *P* values<0.05 were considered significant.

## Results

### Clinicopathological Features

The forty-four patients ranged in age from 24 to 66 years with a median age of 41 years. At the time of surgery, 18 patients (40.9%, 18/44) were classified as stage IB1, 12 (27.3%, 12/44) as stage IB2, 6 (13.6%, 6/44) as stage IIA, 5 (11.4%, 5/44) as stage IIB, 2 (4.5%, 2/44) as stage III and 1 (2.3%, 1/44) as stage IV. Lymph node involvement was present in 14 (31.8%, 14/44) patients at the time of surgery. During the follow-up period, 19 (43.1%, 19/44) patients presented disease recurrence and 13 (29.5%, 13/44) patients died of cervical cancer. The clinicopathological characteristics of the 44 SCCC patients are shown in **Table S1**.

### Reliability of miRNA Detection in FFPET Samples by the All-in-One™ Customized Human qPCR Primer Array

**Figure 1:**
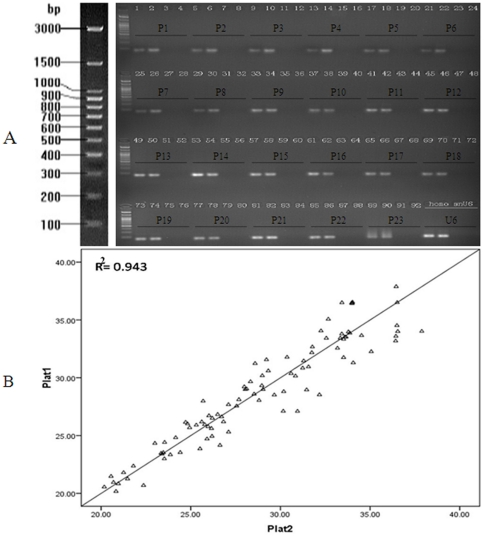
Validation of the All-in-One™ Customized Human qPCR Primer Array detection system. (**A**) Amplification of 30 miRNAs (P1-P30, see Table S2; P24-P30 and SNORD44 are not show in this figure because of limit lane on a single agarose gel; lane1: FFPET human cervix, lane2: frozen human cervix, lane3: RT-minus control and lane4: negative control for each miRNA) and two reference genes (*RNU6B, SNORD44*) from cDNA synthesized from human cervix total RNA. All qPCR products were detected by electrophoresis on a 2.5% agarose gel, indicating a single product of the correct size for each target gene. (**B**) A significant correlation between the miRNA expression patterns in cDNA synthesized from formalin-fixed paraffin-embedded small cell carcinoma of the cervix samples (plat1: sample 1, sample 2, sample 3 and plat2: sample 1, sample 2, sample 3) was observed, R^2^ = 0.943, demonstrating a high reproducibility and reliable detection of miRNA expression levels.

**Figure 2:**
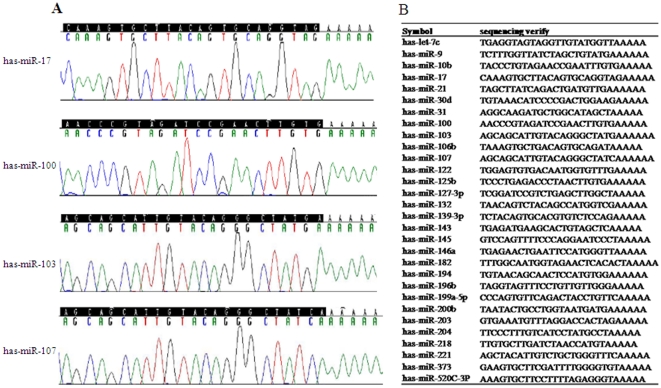
All PCR amplification products were confirmed by sequencing. (A): representative chromas of the 30 miRNAs. (B): the result of sequencing for each miRNAs.

Each primer set cross-linked to the All-in-One qPCR Primer Array was validated to amplify a single product of the correct size for each target gene from microRNAs isolated from human formalin-fixed paraffin-embedded and frozen cervix tissue (**Figure 1A**). The peak values of the amplification and melting curves indicated a single amplification product was obtained in each reaction (data not shown). A significant correlation was observed in the miRNA expression pattern of plat1 (SCCC FFPET sample 1, sample 2, sample 3) and plat2 (SCCC FFPET sample 1, sample 2, sample 3), with an R^2^ value of 0.943(**Figure 1B**). In addition, all PCR amplification products were confirmed by sequencing (**Figure 2**).Taken together, these results indicate that the All-in-One™ Customized Human qPCR Primer Array has a high specificity and sensitivity to detect miRNA expression levels in SCCC FFPET samples, with a high degree of reproducibility.

### Differentially Expressed miRNAs are Associated with Advanced Tumor Stage and Lymph Node Metastasis in SCCC Specimens

**Figure 3:**
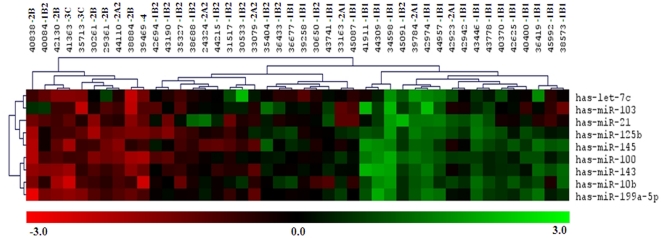
Unsupervised hierarchical cluster analysis of nine differentially expressed miRNAs in 44 samples of advanced and early stage small cell carcinoma of the cervix. Each row represents an individual miRNA and each column represents an individual tissue sample. Two major cluster branches were created, one including mainly advanced tumor stage samples and the other including mainly early tumor stage samples. Red, black, and green pseudocolors indicate transcripts levels below, equal, or above the mean, respectively, on a scale representing gene expression ratios from −3 to 3 on a log 2 scale.

From the 44 SCCC cases, FIGOI-IIA samples were combined as an early stage SCCC group and FIGOIIB-IV samples as an advanced stage SCCC group. Using the GeneCopoeia online Data Analysis System, we identified 9 miRNAs which could significantly discriminate between tumor tissues from the advanced stage SCCC group and early stage SCCC group (*P*<0.05, **Table S2**). Has-let-7c, has-miR-10b, has-miR-100, has-miR-125b, has-miR-143, has-miR-145 and has-miR-199a-5p were significantly down- regulated in advanced stage SCCC, compared to early stage SCCC. Unsupervised hierarchical cluster analysis of the nine differentially expressed miRNAs in all 44 SCCC samples (**Figure 3**) created two major cluster branches, one of which included mainly advanced stage SCCC samples and the other included mainly early stage SCCC samples.

We also identified nine miRNAs (has-let-7c, has-miR-31, has-miR-100, has-miR-125b, has-miR-143, has-miR-145, has-miR-199a-5p, has-miR-203 and has-miR-218) which could significantly discriminate between tumor tissues from patients with metastasis (M, n = 13) and without metastasis (NM, n = 31, *P*<0.05). Interestingly, of the nine miRNAs associated with metastasis, downregulation of has-let-7c, has-miR-100, has-miR-125b, has-miR-143, has-miR-145 and has-miR-199a-5p were also significantly correlated with advanced tumor stage as described above.

### Relationship Between miRNA Expression and SCCC Patient Survival

**Figure 4:**
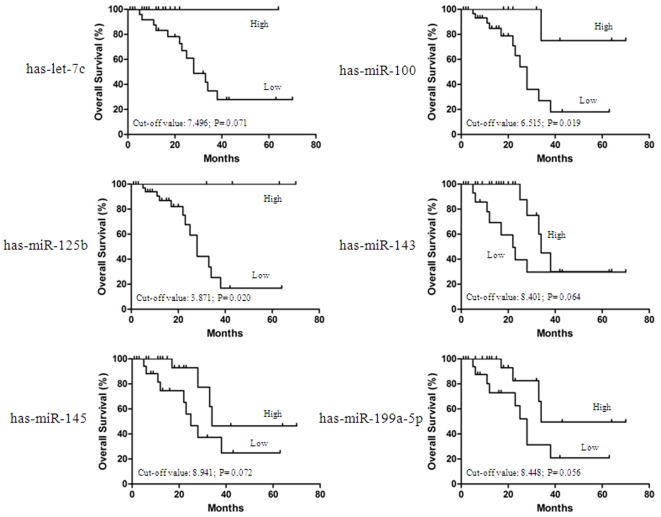
Kaplan-Meier estimates of overall survival in 44 patients with stage small cell carcinoma of the cervix according to has-let-7c (A), has-miR-100 (B), has-miR-125b (C), has-miR-143 (D), has-miR-145 (E) andas-miR-199a-5p expression (F). Has-miR-100 and has-miR-125b down-regulation were significantly associated with poorer survival, and has-let-7c, has-miR-143, has-miR-145 and has-miR-199a-5p down-regulation tended to predict poorer survival, although these results were not statistically significant.

Kaplan–Meier survival analyses revealed that the SCCC patients with low expression of has-miR-100 (*P* = 0.019) and has-miR-125b (*P* = 0.020) had a poorer prognosis compared to patients with high expression of these miRNAs, while has-let-7c (*P* = 0.071), has-miR-143 (*P* = 0.064), has-miR-145 (*P* = 0.072) and has-miR-199a-5p (*P* = 0.056) down-regulation tended to adversely affect survival. However, these findings were not statistically significant (**Figure 4**). Since variables observed to have prognostic influence by univariate analysis may covariate, the downregulation of has-miR-100, has-miR-125b as well as FIGO stage that were significant in univariate analysis were examined in multivariate analysis **(Table S1)**. We found that downregulation of has-miR-100 was evaluated as an independent risk factor for patient survival (hazards ratio: 0.161; 95% confidence interval: 0.036–0.814; *P* = 0.044). Of the other variables, FIGO stage also was found to be independent prognostic predictors for overall survival **(Table S1)**.

### Receiver operating characteristic (ROC) curve analysis

**Figure 5:**
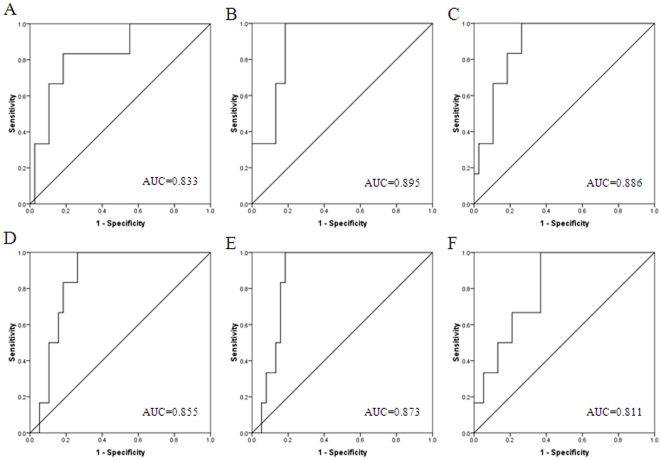
Receiver operating characteristic curve analysis was employed to determine whether the miRNAs found are really good candidates to discriminate early from advanced tumour stages. The sensitivity and specificity for each miRNA were plotted: (A) has-let-7c (*P* = 0.009); (B) has-miR-100 (*P* = 0.002); (C) has-miR-125b (*P* = 0.003); (D) has-miR-143 (*P* = 0.006); (E) has-miR-145 (*P* = 0.004); (F) has-miR-199a-5p (*P* = 0.015).

**Figure 6:**
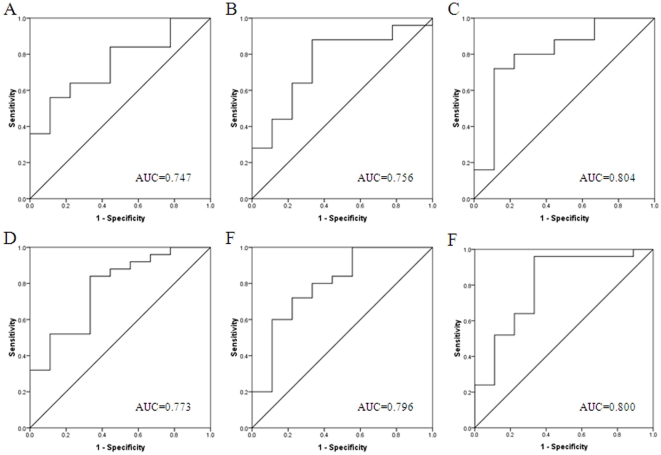
Receiver operating characteristic curve analysis was employed to determine whether the miRNAs found are really good candidates to discriminate the presence or absence of lymph node metastasis. The sensitivity and specificity for each miRNA were plotted: (A) has-let-7c (*P* = 0.030); (B) has-miR-100 (*P* = 0.025); (C) has-miR-125b (*P* = 0.007); (D) has-miR-143 (*P* = 0.016); (E) has-miR-145 (*P* = 0.009); (F) has-miR-199a-5p (*P* = 0.008).

ROC curve analysis showed that the miRNAs found in this study are good candidates to discriminate early from advanced tumour stages and presence or absence of lymph node metastasis. The sensitivity and specificity of discriminate early from advanced tumour stages for each miRNA were plotted: has-let-7c (*P* = 0.009), has-miR-100 (*P* = 0.002), has-miR-125b (*P* = 0.003), has-miR-143 (*P* = 0.006), has-miR-145 (*P* = 0.004), has-miR-199a-5p (*P* = 0.015). Similarly, the sensitivity and specificity of discriminate presence or absence of lymph node metastasis for each miRNA were: has-let-7c (*P* = 0.030, has-miR-100 (*P* = 0.025), has-miR-125b (*P* = 0.007), has-miR-143 (*P* = 0.016), has-miR-145 (*P* = 0.009), has-miR-199a-5p (*P* = 0.008). The corresponding area under the curve (AUC) was collected and shown in Figure 5 and Figure 6.

## Discussion

Small cell carcinoma of the uterine cervix is an uncommon malignancy, accounting for 0.5–3% of all cervical cancers. Similar to lung small cell cancer, SCCC is difficult to manage and usually follows an aggressive clinical course, with death occurring a few years after diagnosis. During the follow-up period of this study, 19 (43.1%) patients presented disease recurrence and 13 (29.5%) patients died. The Gynecologic Oncology Group attempted to study SCCC, but failed to recruit a sufficient number of patients. As a result, treatment decisions in SCCC are based on small single institution studies, and have extrapolated treatment approaches from the management of small cell cancer of the lung. The prognosis of SCCC is considered to be similarly poor to small cell cancer of the lung; however, long-term survival has been reported in SCCC patients [19]–[21]. To determine whether microRNA expression profiles can predict the clinical outcome of SCCC, a total of 44 patients with small cell carcinoma of the cervix were enrolled in this study. Using real-time RT-PCR, we quantified the microRNA expression in FFPET samples of all 44 SCCC patients, and to our knowledge, this is the first attempt to study microRNA molecular markers in SCCC.

Nelson et al. first reported that miRNAs could be isolated and profiled from FFPET samples [22], and subsequently, many studies have reported that small RNA molecules are only slightly affected by tissue processing and that miRNAs demonstrate stable expression in FFPET samples [23]. Our results confirm that miRNAs can be reliably detected in total RNA isolated from SCCC FFPET samples using real-time RT-PCR. The qPCR Primer Array used in this study combines the advantages of real-time RT-PCR technology with a microchip platform to allow simultaneous detection of many genes, ensuring fast, sensitive, quantitative results to allow rapid and accurate screening of interesting genes for further study.

In this study, we observed that downregulation of six miRNAs (has-let-7c, has-miR-100, has-miR-125b, has-miR-143, has-miR-145 and has-miR-199a-5p) is associated with advanced tumor stage, lymph node metastasis and poorer survival in SCCC patients **(** **Table 1** **)**, suggesting that clustering analysis based on miRNA expression may facilitate a detailed individual diagnosis of SCCC patients. Downregulation of miR-143 and 145 are also observed in other tumor types including breast, gastric, liver, lung, bladder, pituitary, ovary and colon cancer [24]–[31]. Several miR-143 targets, including *DNMT3A* and *KRAS* [32], [33], and miR-145 targets including *BNIP3*, *IRS*, *C-MYC*, *YES* and *STAT1* [34]–[36], have been identified, indicating that miR-143 and 145 act as tumor suppressors to repress tumor proliferation or promote apoptosis. Recently, altered processing of miR-143 and 145 had been linked to metastasis [37]–[39], and our study suggests that down-regulation of miR-143 and 145 may also promote lymph node metastasis in SCCC. Sachdeva et al. observed that miR-145 targets multiple metastasis-related genes including *MMP-11* and *ADAM-17* [40], which may potentially contribute to increased metastasis in SCCC.

**Table 1:** Summary of the Associations of Six miRNAs with Advanced Tumor Stage, Lymph Node Metastasis, and Poor Survival in Small Cell Carcinoma of the Cervix.

miRNA	Expressed	Tumor-related	Host Targets
has-let-7c	down	lung and cervical cancer	HMGA2
has-miR-100	down	myeloid leukemia,cervical and prostate cancer	RPSP3,PLK1
has-miR-125b	down	breast and colorectal cancer	BAK1
has-miR-143	down	rectal,colon, liver and lung cancer,leukemia, osteosarcoma	BCL-2,KRAS, DNMT3A
has-miR-145	down	gastric,breast and colon cancer	BNIP3,IRS,STAT1
		squamous cell cancer,bladder tumor	YES,C-MYC
has-miR-199a-5p	down	esophageal adenocarcinoma,testicular tumor, hepatocellular and lung cancer	SWI,SNF,PAK4

Data derived from the NCBI database (http://www.ncbi.nlm.nih.gov/pubmed/).

MiR-199a is downregulated in lung cancer and is suggested to be candidate tumor suppressor [41]. Shen et al. reported that decreased expression of miR-199a-5p contributes to increased cell invasion by functional deregulation of *DDR1* activity in hepatocellular carcinoma [42]. Recently, Hou, et al. observed that miR-199a is frequently downregulated in hepatocellular carcinoma, and that the degree of downregulation significantly correlated with survival, indicating that miR-199a has potential as marker of prognosis in hepatocellular carcinoma [43]. In our study, MiR-199a was downregulated in the SCCC patients with lymph node metastasis compared to patients without metastasis (fold change: 4.774, *P* = 0.004). Conversely, increased expression of miR-199a has been associated with poorer survival in several other tumor types, including acute myeloid leukemia and lung cancer [41], [44], suggesting that the role of miR-199a is dependent on the cell type. Alternatively, cancer cells may produce miR-199a to promote cell migration and invasion, and miR-199a could be downregulated after metastasis. Further study is required to clarify the role of miR-199a in SCCC. As several other miRNAs, including has-let-7c, has-miR-100 and has-miR-125b are also downregulated in SCCC, we aim to determine whether detection and analysis of combined miRNA profiles can determine the prognosis of SCCC patients more precisely in future studies.

In conclusion, this study has revealed that downregulation of has-let-7c, has-miR-100, has-miR-125b, has-miR-143, has-miR-145 and has-miR-199a-5p are significantly correlated with advanced tumor stage, lymph node metastasis and poorer survival in SCCC. Identification of altered expression of specific miRNAs may further elucidate the steps in the SCCC metastatic pathway and allow for development of novel targeted therapies. Although this study has a simple design and is limited by the small numbers of patients, it is the largest series of SCCC patients reported to date. We hope that identification of the microRNA profile in SCC contributes to an improved diagnostic and prognostic ability for this rare and aggressive tumor.

## Supporting Information

Table S1 Univariate and multivariate analysis of patient survival based on clinical and pathologic factors and miRNAs.
(DOCX)

Table S2 MicroRNA Fold Changes in Small Cell Carcinoma of the Cerxix Detected Using a qPCR Primer Array Detection System.
(DOCX)
