# Supplementary material for: Downregulation of Six MicroRNAs Is Associated with Advanced Stage, Lymph Node Metastasis and Poor Prognosis in Small Cell Carcinoma of the Cervix
Source: PLoS One. 2012 Mar 16;7(3):e33762. doi: 10.1371/journal.pone.0033762 (PMC3306296; doi:10.1371/journal.pone.0033762)
Supplement: Table S1 — Univariate and multivariate analysis of patient survival based on clinical and pathologic factors and miRNAs. (DOCX) [file pone.0033762.s001.docx]

| **Table S1.** | | | | | | | | |
| --- | --- | --- | --- | --- | --- | --- | --- | --- |
| **Variables** | **Number** | **Percent (%)** |  | **Univariate analysis ^*^** | |  | **Multivariate analysis ^&^** | |
|  |  |  |  | **3-year OS(%)** | **P Value** |  | **Hazards ratio (95% CI)** | **P Value** |
|  |  |  |  |  |  |  |  |  |
| **Age(years)** |  |  |  |  |  |  |  |  |
| <40 | 16 | 36.4 |  | 48.4 |  |  |  |  |
| ≥40 | 28 | 63.6 |  | 36.0 | 0.522 |  |  |  |
| **FIGO stage** |  |  |  |  |  |  |  |  |
| Ⅰb1 | 18 | 40.9 |  | 75.0 |  |  |  |  |
| Ⅰb2-Ⅳ | 26 | 59.1 |  | 11.4 | **<0.0001** |  | 5.538 (3.321-13.1120) | **0.026** |
| **Lymph node metastasis** |  |  |  |  |  |  |  |  |
| Positive | 14 | 31.8 |  | 33.4 |  |  |  |  |
| Negative | 30 | 68.2 |  | 53.6 | 0.340 |  |  |  |
| **Tumor mass size** |  |  |  |  |  |  |  |  |
| <4cm | 28 | 63.6 |  | 47.0 |  |  |  |  |
| ≥4cm | 16 | 36.4 |  | 23.2 | 0.474 |  |  |  |
| **Lymph-vascular space invasion** |  |  |  |  |  |  |  |  |
| Positive | 13 | 29.5 |  | 23.9 |  |  |  |  |
| Negative | 31 | 70.5 |  | 61.1 | 0.082 |  |  |  |
| **Depth of stromal invasion** |  |  |  |  |  |  |  |  |
| <2/3 | 35 | 79.5 |  | 46.5 |  |  |  |  |
| ≥2/3 | 9 | 20.5 |  | 26.0 | 0.565 |  |  |  |
| **Neurone-specific enolase** |  |  |  |  |  |  |  |  |
| Positive | 35 | 94.6 |  | 32.3 |  |  |  |  |
| Negative | 2 | 5.4 |  | 100.0 | 0.683 |  |  |  |
| **Synaptophysin** |  |  |  |  |  |  |  |  |
| Positive | 27 | 84.3 |  | 35.9 |  |  |  |  |
| Negative | 5 | 15.7 |  | 75.0 | 0.662 |  |  |  |
| **has-let-7c** |  |  |  |  |  |  |  |  |
| High | 14 | 31.8 |  | 100.0 |  |  |  |  |
| Low | 30 | 69.2 |  | 34.8 | 0.071 |  |  |  |
| **has-miR-100** |  |  |  |  |  |  |  |  |
| High | 10 | 22.7 |  | 75.0 |  |  |  |  |
| Low | 34 | 77.3 |  | 27.0 | **0.019** |  | 0.161 (0.036-0.814) | **0.044** |
| **has-miR-125b** |  |  |  |  |  |  |  |  |
| High | 4 | 9.1 |  | 100.0 |  |  |  |  |
| Low | 40 | 90.9 |  | 25.4 | **0.020** |  | 0.352 (0.102-1.014) | **0.057** |
| **has-miR-143** |  |  |  |  |  |  |  |  |
| High | 30 | 69.2 |  | 45.0 |  |  |  |  |
| Low | 14 | 31.8 |  | 29.7 | 0.064 |  |  |  |
| **has-miR-145** |  |  |  |  |  |  |  |  |
| High | 26 | 59.1 |  | 46.4 |  |  |  |  |
| Low | 18 | 40.9 |  | 37.3 | 0.072 |  |  |  |
| **has-miR-199a-5p** |  |  |  |  |  |  |  |  |
| High | 24 | 54.5 |  | 49.5 |  |  |  |  |
| Low | 20 | 45.5 |  | 31.3 | 0.056 |  |  |  |
| **CI:** confidence interval; *****Log-rank; **&**Cox regression model; Signiﬁcant values are in **bold**. | | | | | | | | |
