# Supplementary material for: Downregulation of Six MicroRNAs Is Associated with Advanced Stage, Lymph Node Metastasis and Poor Prognosis in Small Cell Carcinoma of the Cervix
Source: PLoS One. 2012 Mar 16;7(3):e33762. doi: 10.1371/journal.pone.0033762 (PMC3306296; doi:10.1371/journal.pone.0033762)
Supplement: Table S2 — MicroRNA Fold Changes in Small Cell Carcinoma of the Cerxix Detected Using a qPCR Primer Array Detection System. (DOCX) [file pone.0033762.s002.docx]

| **Table S2.** | | | | | | | | | | |
| --- | --- | --- | --- | --- | --- | --- | --- | --- | --- | --- |
| **No.** | **Symbol** | **FIGO Stage** | | | |  | **Lymph Node Metastasis** | | | |
|  |  | **ⅠB1^＊^** | **ⅠB2-Ⅳ^＊^** | **Fold Change^§^** | **P Value^※^** |  | **Yes^＊^** | **No^＊^** | **Fold Change^§^** | **P Value^※^** |
| **1** | **has-let-7c** | 25.388 | 26.236 | 0.224 | **0.014** |  | 23.457 | 25.662 | 2.643 | **0.019** |
| **2** | **has-miR-9** | 31.226 | 30.882 | 0.516 | 0.411 |  | 30.924 | 31.090 | 0.648 | 0.536 |
| **3** | **has-miR-10b** | 29.878 | 31.296 | 0.168 | **0.005** |  | 28.250 | 30.211 | 2.180 | 0.095 |
| **4** | **has-miR-17** | 28.667 | 27.823 | 0.632 | 0.435 |  | 27.768 | 28.458 | 0.924 | 0.891 |
| **5** | **has-miR-21** | 24.085 | 25.169 | 0.242 | 0.069 |  | 23.152 | 24.222 | 1.203 | 0.755 |
| **6** | **has-miR-30d** | 30.724 | 31.242 | 0.270 | 0.153 |  | 28.779 | 30.750 | 2.191 | 0.256 |
| **7** | **has-miR-31** | 32.603 | 34.658 | 0.591 | 0.560 |  | 30.842 | 33.991 | 5.011 | **0.019** |
| **8** | **has-miR-100** | 27.337 | 30.245 | 0.034 | **0.001** |  | 24.766 | 28.048 | 5.438 | **0.017** |
| **9** | **has-miR-103** | 26.789 | 27.081 | 0.368 | 0.061 |  | 25.274 | 26.902 | 1.772 | 0.162 |
| **10** | **has-miR-106b** | 30.282 | 30.715 | 0.664 | 0.407 |  | 29.291 | 30.812 | 1.562 | 0.306 |
| **11** | **has-miR-107** | 32.108 | 31.670 | 1.122 | 0.852 |  | 31.325 | 31.922 | 0.866 | 0.795 |
| **12** | **has-miR-122** | 34.765 | 34.917 | 1.492 | 0.519 |  | 34.209 | 35.336 | 1.026 | 0.958 |
| **13** | **has-miR-125b** | 22.405 | 25.143 | 0.073 | **0.006** |  | 19.973 | 23.289 | 5.712 | **0.007** |
| **14** | **has-miR-127-3p** | 25.727 | 26.369 | 1.347 | 0.653 |  | 24.832 | 26.162 | 1.442 | 0.478 |
| **15** | **has-miR-132** | 30.401 | 31.102 | 0.487 | 0.218 |  | 29.629 | 30.714 | 1.216 | 0.680 |
| **16** | **has-miR-139-3p** | 30.968 | 31.802 | 1.074 | 0.929 |  | 29.794 | 31.364 | 1.702 | 0.379 |
| **17** | **has-miR-143** | 26.875 | 28.691 | 0.113 | **0.015** |  | 24.233 | 27.504 | 5.537 | **0.010** |
| **18** | **has-miR-145** | 23.183 | 25.689 | 0.085 | **0.012** |  | 20.386 | 24.216 | 8.154 | **0.006** |
| **19** | **has-miR-146a** | 32.395 | 32.782 | 0.744 | 0.748 |  | 30.579 | 32.627 | 2.191 | 0.280 |
| **20** | **has-miR-182** | 32.111 | 32.079 | 0.569 | 0.464 |  | 31.561 | 32.250 | 0.782 | 0.711 |
| **21** | **has-miR-194** | 32.990 | 32.840 | 0.927 | 0.937 |  | 32.405 | 33.028 | 0.953 | 0.953 |
| **22** | **has-miR-196b** | 31.129 | 33.386 | 0.251 | 0.080 |  | 29.908 | 31.690 | 2.106 | 0.267 |
| **23** | **has-miR-199a-5p** | 29.254 | 30.818 | 0.179 | **0.023** |  | 26.860 | 30.000 | 4.774 | **0.004** |
| **24** | **has-miR-200b** | 28.206 | 28.364 | 0.301 | 0.235 |  | 28.022 | 27.804 | 0.504 | 0.458 |
| **25** | **has-miR-203** | 32.403 | 34.506 | 0.850 | 0.890 |  | 29.813 | 33.540 | 7.651 | **0.031** |
| **26** | **has-miR-204** | 31.874 | 32.916 | 1.177 | 0.878 |  | 30.702 | 31.789 | 0.860 | 0.872 |
| **27** | **has-miR-218** | 32.711 | 34.222 | 0.378 | 0.234 |  | 30.388 | 33.406 | 4.611 | **0.015** |
| **28** | **has-miR-221** | 30.013 | 30.918 | 1.025 | 0.975 |  | 28.792 | 30.191 | 1.776 | 0.339 |
| **29** | **has-miR-520C-3P** | 34.785 | 34.237 | 0.863 | 0.935 |  | 24.520 | 32.315 | 16.524 | 0.092 |
| **30** | **has-miR-373** | 35.263 | 34.849 | 3.085 | 0.090 |  | 35.547 | 35.396 | 0.519 | 0.219 |
| **＊: Threshold Cycle(Ct); §: 2^-ΔΔCt; ※: T test; S**igniﬁcant values are in **bold**. | | | | | | | | | | |
